# Distinct Roles of SOM and VIP Interneurons during Cortical Up States

**DOI:** 10.3389/fncir.2016.00052

**Published:** 2016-07-26

**Authors:** Garrett T. Neske, Barry W. Connors

**Affiliations:** Department of Neuroscience, Division of Biology and Medicine, Brown University, ProvidenceRI, USA

**Keywords:** cortex, interneuron, Up state, somatostatin, VIP

## Abstract

During cortical network activity, recurrent synaptic excitation among pyramidal neurons is approximately balanced by synaptic inhibition, which is provided by a vast diversity of inhibitory interneurons. The relative contributions of different interneuron subtypes to inhibitory tone during cortical network activity is not well-understood. We previously showed that many of the major interneuron subtypes in mouse barrel cortex are highly active during Up states (Neske et al., 2015); while fast-spiking (FS), parvalbumin (PV)-positive cells were the most active interneuron subtype, many non-fast-spiking (NFS), PV-negative interneurons were as active or more active than neighboring pyramidal cells. This suggests that the NFS cells could play a role in maintaining or modulating Up states. Here, using optogenetic techniques, we further dissected the functional roles during Up states of two major NFS, PV-negative interneuron subtypes: somatostatin (SOM)-positive cells and vasoactive intestinal peptide (VIP)-positive cells. We found that while pyramidal cell excitability during Up states significantly increased when SOM cells were optogenetically silenced, VIP cells did not influence pyramidal cell excitability either upon optogenetic silencing or activation. VIP cells failed to contribute to Up states despite their ability to inhibit SOM cells strongly. We suggest that the contribution of VIP cells to the excitability of pyramidal cells may vary with cortical state.

## Introduction

Neocortical inhibitory interneurons provide indispensable balance to the strong recurrent excitation within the pyramidal cell population. Inhibitory interneurons are notoriously diverse in their intrinsic physiology, synaptic targeting patterns, and short-term synaptic dynamics (Markram et al., 2004; Fishell and Rudy, 2011; Gentet, 2012). Furthermore, interneurons target not only pyramidal cells, but also other interneurons, often in quite selective ways (Beierlein et al., 2003; Pfeffer et al., 2013; Karnani et al., 2016b). State-dependent neuromodulation of intrinsic excitability or synaptic release can also vary among interneuron subtypes (Muñoz and Rudy, 2014). Thus, it is difficult to predict the precise contribution of different interneuron subtypes to the overall inhibitory conductance in pyramidal cells during cortical network activity.

During periods of behavioral quiescence, such as during slow-wave sleep and under certain anesthetics, the cortical network undergoes a slow (<1 Hz) oscillation between periods of vigorous synaptic activity (Up states) and marked silence (Down states; Steriade et al., 1993; Chauvette et al., 2010; Neske, 2016). The essential neuronal machinery for the generation of the slow oscillation is available in local cortical circuits since such activity can be observed in isolated cortical slabs *in vivo* (Timofeev et al., 2000) and in cortical slices *in vitro* (Sanchez-Vives and McCormick, 2000). Cortical Up states themselves share many features of the waking, “activated” cortex (Destexhe et al., 2007) and the variable synaptic barrages associated with gain modulation in active cortical processing (Haider and McCormick, 2009). Thus, studying the cellular and network properties of Up states is relevant not only for understanding the dynamics of the quiescent cortex, but perhaps also for the moment-to-moment fluctuations inherent to the cortex in the waking, information-processing state.

We have previously shown that in mouse barrel cortex *in vitro*, all of the major neurochemically defined interneuron subtypes exhibit spiking activity during Up states, though to different degrees (Neske et al., 2015). While fast-spiking (FS), parvalbumin (PV)-positive interneurons were by far the most active interneuron subtype, two of the major non-fast-spiking (NFS) PV-negative subtypes were as active or more active than neighboring pyramidal cells. These NFS, PV-negative interneurons were somatostatin (SOM)-positive cells and vasoactive-intestinal peptide (VIP)-positive cells. Our results suggested that while PV cells likely provide the majority of the inhibitory regulation of the Up state, SOM and VIP cells also have the potential to contribute to this regulation.

Somatostatin and VIP cells have distinctive synaptic targeting properties. While most SOM cells target the distal apical dendrites of pyramidal cells (Wang et al., 2004; Silberberg and Markram, 2007), VIP cells selectively innervate SOM cells (Lee et al., 2013; Pfeffer et al., 2013; Pi et al., 2013; Karnani et al., 2016a,b). In different scenarios involving activation of VIP cells in awake animals, the result is disinhibition of pyramidal cells (Pi et al., 2013; Fu et al., 2014; Reimer et al., 2014; Zhang et al., 2014; Karnani et al., 2016a).

Here, using optogenetic techniques, we determined that spontaneous SOM cell activity during Up states significantly controls the spike output of pyramidal cells. Spontaneous VIP cell activity, however, did not affect the spike output of pyramidal cells. Moreover, strong activation of VIP cells during Up states also did not affect the spike output of pyramidal cells. We suggest several possible reasons why VIP cells may be ineffective in controlling the spike output of pyramidal cells during Up states.

## Materials and Methods

All procedures involving laboratory animals were approved by the Brown University Institutional Animal Care and Use Committee.

### Mouse Lines

To express optogenetic actuators in SOM and VIP cells, we crossed either homozygous SOM-Cre mice (STOCK *Sst^tm2.2(cre)Zjh^*/J, The Jackson Laboratory) or homozygous VIP-Cre mice (STOCK *Vip^tm1(cre)Zjh^/*J, The Jackson Laboratory) with homozygous archaerhodopsin (Arch) reporter mice (B6; 129S -*Gt(ROSA)26Sor^tm35.1(CAG-aop3/GFP)Hze^/*J, The Jackson Labo ratory) for optogenetic silencing, or with homozygous channelrhodopsin (ChR2) reporter mice (B6.Cg-*26Sor^tm32(CAG-COP4∗H134R/EY FP)Hze^/*J, The Jackson Laboratory) for optogenetic activation. To target either PV cells or SOM cells for recording while optogenetically activating VIP cells, we crossed either of two homozygous transgenic interneuron GFP lines with the homozygous VIP-Cre line. These transgenic interneuron GFP lines were: the GIN line [FVB-Tg(GadGFP)45704Swn/J], which expresses GFP in a subset of SOM cells (Oliva et al., 2000), particularly in cortical layer 2/3, and the G42 line [CB6-Tg(Gad1-EGFP)G42Zjh/J], which expresses GFP in a subset of PV cells (Chattopadhyaya et al., 2004), also particularly in cortical layer 2/3. Not all SOM and PV cells are fluorescently labeled in the GIN and G42 lines, respectively.

### Viral Injections

In progeny resulting from crosses of either the VIP-Cre line and the GIN line or the VIP-Cre line and the G42 line (see Mouse Lines), ChR2 was introduced into VIP cells via stereotaxic viral injection into barrel cortex. Adeno-associated virus (AAV) carrying a genetic construct for RFP-tagged ChR2 was used: AAV2/1Ef1α.DIO.hChRr2(H134R)-mCherry.WPRE.hGH (Penn Vector Core). To minimize toxicity, virus used for injections was diluted with sterile saline from an initial titer of 4.5 × 10^12^ IU/mL to a final titer of 4.5 × 10^11^ IU/mL.

For the surgical procedure involving stereotaxic viral injection, mice aged ∼P10 were injected intraperitoneally with a ketamine (70 mg/kg)/Dormitor (0.25 mg/kg)/saline anesthesia cocktail. After waiting 10 min and checking for a pain response by a tail pinch, mice were placed in a stereotaxic frame and the scalp was opened to perform a craniotomy at (in mm) +3.4 (x) and -0.8 (y) from bregma. Virus (∼1.5 μL) was drawn into a glass micropipette. This micropipette was then lowered slowly (∼1 mm/min) through the craniotomy into barrel cortex, 0.4 mm from the pial surface (approximately targeting cortical layer 3). Virus was then pressure-ejected with a Picospritzer over the course of ∼30 min. After injection of the virus, the micropipette was left in place for ∼5 min before slowly removing it from the cortex. After closing the scalp with Gluture, mice were awoken with Antisedan and allowed to recover on a heating pad for ∼1 h before being returned to their home cage. Virus was allowed to express 8 days before experiments.

### Slice Preparation

Slices (320–400 μm) were prepared from mouse barrel cortex (mice of both sexes aged P14-P19). Mice were first anesthetized via inhalation of isofluorane, then decapitated with a scalpel blade, after which the head was transferred to ice-cold (∼0°C) artificial CSF (ACSF) saturated with a 95% O_2_/5% CO_2_ mixture for brain extraction. The composition of the ACSF was as follows (in mM): 126 NaCl, 3 KCl, 10 dextrose, 26 NaHCO_3_, 1.25 NaH_2_PO_4_, 2 CaCl_2_, and 2 MgSO_4_. A mid-sagittal cut was made, and one hemisphere was glued on an angled block to cut slices in the thalamocortical plane for barrel cortex (Agmon and Connors, 1991) with a Leica VT 1000S vibratome. ACSF temperature was ∼0°C throughout slicing. After slices were cut, they were immediately transferred to a submerged holding chamber maintained at ∼33°C containing ACSF. The slices remained at this temperature and in this ACSF for 30 min before the chamber was cooled to room temperature (∼24°C). Slices remained at this temperature until use in the recording chamber.

### Electrophysiological Recordings

Slices were transferred to a recording chamber in which ACSF bathed both sides of the slice (Warner Instruments, Model RC-27L). To promote Up and Down states, the flow rate of ACSF was kept high (∼10 mL/min) to ensure ample oxygenation of the tissue (Hájos et al., 2004; Hájos and Mody, 2009). The temperature of the solution in the recording chamber was 33°C. Between recording sessions, slices were superfused with ACSF of the same composition as the solution in the holding chamber. During recordings of Up and Down states, the ACSF was changed to (in mM): 126 NaCl, 5 KCl, 20 dextrose, 26 NaHCO_3_, 1.25 NaH_2_PO_4_, 1 CaCl_2_, and 1 MgSO_4_. Spontaneous Up and Down states usually occurred within 1 min of changing the superfusing solution to this “modified ACSF.”

For this study, we focused exclusively on electrically evoked Up states so that optical stimulation could be timed precisely relative to Up state onset (see Optogenetics). We evoked Up states electrically with a twisted bipolar electrode (FHC) in layer 5, controlled by a stimulus isolation unit (Cygnus SIU-91). Digital command of the SIU was provided by a Cygnus PG4000 digital stimulator, which was triggered manually. Manual triggering was used in order to visually monitor spontaneous Up states so that they did not interfere with evoked Up states. When a spontaneous Up state occurred, we waited at least 5 s before manually triggering an evoked Up state. This waiting period was sufficient to bypass the network refractory period, because spontaneous Up states could often occur within this 5 s window. Electrical stimulation was of the lowest intensity that reliably evoked Up states (5–30 μA, pulse width 400 μs). Our previous work (Neske et al., 2015) and the work of others (Shu et al., 2003) suggest that Up states evoked by low-intensity intracortical electrical stimulation are virtually indistinguishable from spontaneous Up states. Thus, studying Up states evoked in this way can justifiably be considered equivalent to studying Up states occurring spontaneously. Examples of spontaneous Up states from the cell types we study here can be found in Neske et al. (2015).

Cells were visualized with an Olympus BX50WI microscope equipped with DIC and epifluorescence optics. Pyramidal cells were targeted for recording based on their characteristic appearance with DIC optics (i.e., upward somatic taper and apical dendrite) and verified *post hoc* by their regular-spiking (RS) physiology, while opsin-expressing cells (i.e., VIP or SOM cells) and transgenic-GFP-expressing cells (i.e., GIN or G42 cells) were targeted based on their fluorescence.

Whole-cell recordings were performed with borosilicate glass pipettes pulled to final tip resistances between 4 and 7 MΩ. For current-clamp recordings, micropipettes were filled with internal solution of the following composition (in mM): 130 K gluconate, 4 KCl, 2 NaCl, 10 HEPES, 0.2 EGTA, 4 ATP-Mg, 0.3 GTP-Na, and 14 phosphocreatine-2K. For voltage-clamp recordings of GIN, G42, and pyramidal cells (see VIP Cells Strongly Inhibit SOM Cells in Layer 2/3 Barrel Cortex), micropipettes were filled with (in mM): 130 Cs gluconate, 4 CsCl, 2 NaCl, 10 HEPES, 0.2 EGTA, 4 ATP-Mg, 0.3 GTP-Na, 14 phosphocreatine-2Na, and 5 QX-314. Internal solutions had a final osmolality of 290–295 mOsm and pH of 7.22–7.25. Recordings were made with a MultiClamp 700B patch-clamp amplifier (Axon), in which signals were first filtered (DC–10 kHz) and then digitized at 20 kHz with the Digidata 1440A data acquisition system and Clampex data acquisition software (Axon). Micropipette capacitance was compensated in the bath, and the bridge was balanced after attaining the whole-cell configuration. Cells with bridge-balance values >30 MΩ were not used. For voltage-clamp recordings, series resistance compensation was always performed online, with prediction/correction set between 70 and 80%. Series resistances were continually monitored during experiments to ensure sufficient compensation.

For recordings of VIP-cell-evoked inhibitory post-synaptic currents (IPSCs) in GIN, G42, and pyramidal cells, 50 μM APV and DNQX were added to “modified ACSF” (i.e., that which would promote spontaneous Up states if excitatory transmission were not blocked). Cells were voltage-clamped at 0 mV to isolate the evoked IPSCs. The stimulus evoking the IPSCs was a single, 5-ms light pulse delivered by whole-field illumination through the 40x immersion objective every 30 s (see Optogenetics).

### Optogenetics

For optical stimulation of Arch- or ChR2-expressing cells, collimated light from a white LED (cool white 5500K, Mightex) controlled by a Thorlabs LEDD1B driver was reflected through a dichroic mirror (FF655-Di01, Semrock) and a 40x immersion objective (LUMPlanFl 40x/0.80 W, Olympus). This resulted in a spot size with a radius of ∼270 μm. The maximum possible light power at the focal plane (as measured by a S120C photodiode power sensor coupled to an analog power meter, Thorlabs) was 18.5 mW (measured at 465 nm, for ChR2) and 12.5 mW (measured at 590 nm, for Arch). During recordings, the light spot was centered over the recorded cell. Either long light pulses (∼500 ms pulse width) or trains of short light pulses (40 or 50 Hz, 5 ms pulse width) were commanded by a Cygnus PG4000 digital stimulator, which simultaneously commanded an SIU so that temporal relations between Up state onset and onset of light stimulus could be controlled.

### Data Acquisition and Analysis

The primary data of interest were changes in pyramidal cell firing rates during Up states when different interneuron subtypes were optogenetically silenced or activated, compared to control conditions in which no light stimulus was given. For most recordings, a pyramidal cell was recorded in current-clamp and intracortical electrical stimulation, which evoked an Up state with <10 ms latency, was followed 250 ms later by a long light pulse (for SOM-Arch and VIP-Arch experiments) or a 40 Hz train of 5-ms-long light pulses (for VIP-ChR2 experiments). The rationale for timing the light pulse after Up state onset was to allow recurrent network activity to initiate normally such that we could observe contributions of different interneuron subtypes after network activity had begun. The exact duration of the long light pulse or the 40-Hz pulse train depended on the average evoked Up state duration. In most cases, the duration of the long light pulse or 40 Hz pulse train was ∼500 ms, which allowed light stimuli to be given for most of the Up state but before Up state termination. During recordings, we interleaved single trials with electrical stimulation only and single trials with electrical stimulation plus light stimulation. During a recording, each condition (i.e., electrical alone or electrical plus light) was repeated 20–25 times.

To calculate the Up state firing rates of pyramidal cells during periods with light stimulation and during control periods, we counted the number of spikes that occurred in the temporal window after Up state initiation in which the light stimulus was given and divided this number by the duration of this temporal window. For instance, if a 500-ms-long light stimulus was given 250 ms after Up state onset, spikes would be counted (for both electrical only and electrical plus light trials) from 250 to 750 ms after Up state onset. The trial-averaged Up state firing rate during electrical only and during electrical plus light conditions were considered the average control Up state firing rate and the average Up state firing under optogenetic manipulation for a given pyramidal cell, respectively.

For VIP-Arch and VIP-ChR2 experiments, in addition to timing light stimuli after Up state onset, we also performed experiments in which we timed the light stimuli before Up state onset. The rationale for these additional experiments is discussed in Section “Results.” An additional metric for these experiments was the delay between Up state onset and the time at which the pyramidal cell fired its first spike, which, as for firing rate, was trial-averaged for each condition in each pyramidal cell.

Due to the low Up state firing rates of pyramidal cells in layer 2/3 of barrel cortex (Neske et al., 2015), for most recordings of pyramidal cells in this layer, we injected depolarizing current into the cells to bring them closer to spike threshold during Up states (bringing them to between -60 to -55 mV during the Down state) in order to count enough spikes within the analysis window. This manipulation was not required for layer 5 pyramidal cells. While it could be argued that this manipulation might lead to data that are not relevant to the natural spiking behavior of most layer 2/3 pyramidal cells, there are pyramidal cells in this layer, albeit small in number (yet possibly of particular functional relevance), that exhibit higher firing rates (∼5 Hz) during spontaneous or sensory-evoked depolarizing periods (Yassin et al., 2010; Jouhanneau et al., 2014). Furthermore, depolarizing pyramidal cells moves them further from the GABA_A_ reversal potential, which increases the ability to detect possible effects of inhibitory interneurons on Up state excitability of pyramidal cells.

### Statistics

For statistical comparisons, distributions of data were first tested for normality with a Kolmogorov–Smirnov test. If data were normally distributed, standard parametric statistics were used: unpaired or paired t tests for comparisons of two groups and one-way or repeated-measures ANOVA for comparisons of multiple groups. If data were not normally distributed, non-parametric statistics were used: Mann–Whitney or Wilcoxon’s matched pairs test for comparisons of two groups and Kruskal–Wallis or Friedman’s test for comparisons of multiple groups. Bonferroni correction was used for multiple comparisons. *P*-values < 0.05 were considered significant. All analyses of data were conducted in Mathematica 9 (Wolfram Research). Error bars are SEM, unless otherwise noted.

## Results

Our goal was to study the functional contribution of two types of NFS, PV-negative interneurons (SOM and VIP cells) to the inhibitory modulation of pyramidal cells during Up states. While FS, PV-positive interneurons spike the most during Up states in mouse barrel cortex (Fanselow and Connors, 2010; Neske et al., 2015), SOM and VIP cells are active as well. Thus, SOM and VIP cells have the potential to contribute to the inhibitory modulation of pyramidal cells during Up states, but whether they do so is unclear. To test this possibility, we first performed optogenetic silencing experiments during Up states in barrel cortex, in which either SOM or VIP cells expressed Arch. We also further queried the possible disinhibitory role of VIP cells in optogenetic activation experiments, in which VIP cells expressed ChR2.

### SOM Cells Control Spiking Output of Pyramidal Cells during Up States

**FIGURE 1:**
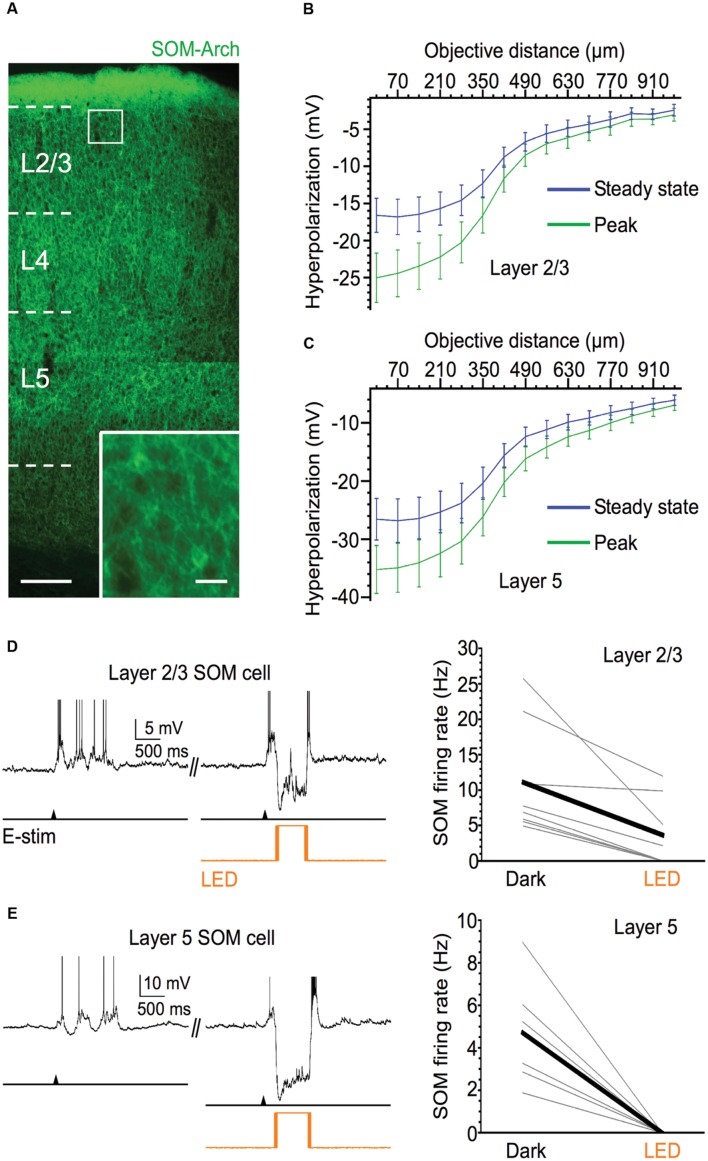
**Efficacy of Arch expression in SOM cells.** **(A)** Fluorescent image of Arch-EYFP-expressing SOM cells throughout barrel cortex; scale bar = 200 μm. Inset: expanded view of 2 Arch-expressing SOM cells, scale bar = 20 μm. **(B)** Light-evoked hyperpolarization (500 ms pulse duration) of Arch-expressing SOM cells in layer 5 as 40x immersion objective is moved away from the recorded cell in 70 μm increments (*n* = 6 cells). Values plotted on green curve are peak values of hyperpolarization and values plotted on blue curve are steady-state values of hyperpolarization (measured during 50-ms period before offset of light stimulus). **(C)** As in **(B)**, but for Arch-expressing SOM cells in layer 2/3 (*n* = 6 cells). **(D)** Light stimuli significantly decrease spiking output of layer 2/3 Arch-expressing SOM cells during evoked Up states. Example voltage trace is shown above. Shown below are population data (*n* = 8 cells; Dark: 11.1 ± 2.8 Hz, LED: 3.6 ± 1.7 Hz, *P* = 0.014, paired *t*-test). **(E)** Light stimuli completely silence Up state spiking in all recorded layer 5 Arch-expressing SOM cells. Example voltage trace is shown above. Shown below are population data (*n* = 6 cells; Dark: 4.7 ± 1.1 Hz, LED: 0 Hz, *P* = 0.007, paired *t*-test). Action potentials are truncated in **(D,E)**.

To test whether SOM cells contribute to the inhibitory modulation of pyramidal cells during Up states, we silenced their spiking activity through Cre-dependent expression of Arch and photostimulation of the SOM cell population in the slice. We tested the contribution of SOM cells to pyramidal cell excitability in layers 5 and 2/3 of barrel cortex. We first ensured that light stimuli were effective in silencing the activity of SOM cells during Up states by recording from these cells directly (**Figure 1**). For SOM cells centered in the light spot, the light-evoked hyperpolarization (measured during Down states) was quite strong (means of layer 5: -35.2 mV at peak, -26.6 mV at steady-state; layer 2/3: -22.9 mV at peak, -15.3 mV at steady-state). To estimate the spatial extent of the light-evoked hyperpolarization of Arch-expressing SOM cells, we moved the 40x immersion objective horizontally (approximately within a cortical layer) in 70 μm increments. While the light-evoked hyperpolarizing responses fell off rapidly after moving the objective a distance approximately equal to the radius of the light spot size (∼270 μm), responses remained sizable after this point, and detectable even at ∼1 mm away from the recorded cell (**Figure 1B**). Thus, while optical silencing of Arch-expressing SOM cells is most effective within ∼270 μm of the recorded cell, a substantial number of SOM cells outside of this radius are likely also to be effectively silenced.

To test the effectiveness of light-evoked hyperpolarizations of Arch-expressing SOM cells, we applied light stimuli while recording from SOM cells engaged in Up states. Photostimulation completely silenced or greatly diminished the spiking activity of SOM cells during Up states (**Figures 1D,E**, Dark vs. LED). In layer 5, photostimulation completely silenced all recorded SOM cells during Up states (from a mean firing rate of 4.7–0 Hz; **Figure 1E**), and in layer 2/3, optical stimulation either completely silenced SOM cells or significantly decreased their spiking output by ≥50% (from a mean firing rate of 11.1–3.6 Hz; **Figure 1D**).

**FIGURE 2:**
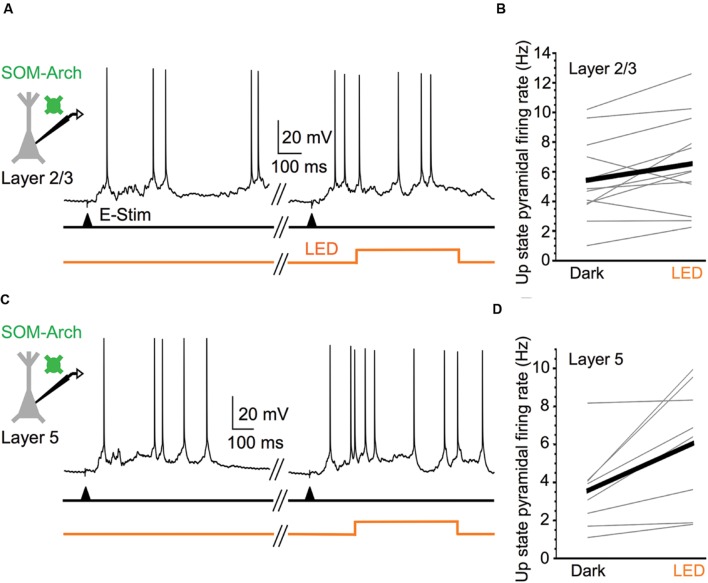
**Silencing SOM cells during Up states increases pyramidal cell excitability.** **(A)** Representative Up state membrane potential trace of a layer 2/3 pyramidal cell with and without optogenetic stimulation of Arch-expressing SOM cells in layer 2/3. **(B)** Population data demonstrating layer 2/3 pyramidal cell Up state firing rates increase when SOM cells are silenced (*n* = 12 cells; Dark: 5.4 ± 0.8 Hz, LED: 6.5 ± 0.9 Hz, *P* = 0.037, paired *t*-test). **(C)** As in **(A)**, but for a representative layer 5 pyramidal cell. **(D)** As in **(B)**, but for layer 5 pyramidal cells (*n* = 8 cells; Dark: 3.6 ± 0.8 Hz, LED: 6.0 ± 1.2 Hz, *P* = 0.018, paired *t*-test).

With the assurance that optical stimulation of Arch-expressing SOM cells powerfully suppresses their activity during Up states, we then considered the effect of silencing SOM cells on the spiking output of pyramidal cells. Comparing the firing rates during the middle portion of Up states in layer 5 and layer 2/3 pyramidal cells, we found that, in both layers, these cells significantly increased their spiking output when SOM cells were silenced compared to control periods (**Figure 2**). SOM cell silencing increased firing rate in the pyramidal cell population in layer 5 from 3.6 to 6.0 Hz and in layer 2/3 from 5.4 to 6.5 Hz. Thus, the virtual removal of SOM cells from the cortical network during Up states significantly enhanced the spiking output of pyramidal cells, suggesting that SOM cells provide functionally important levels of inhibition during spontaneous network activity.

### VIP Cells Strongly Inhibit SOM Cells in Layer 2/3 Barrel Cortex

**FIGURE 3:**
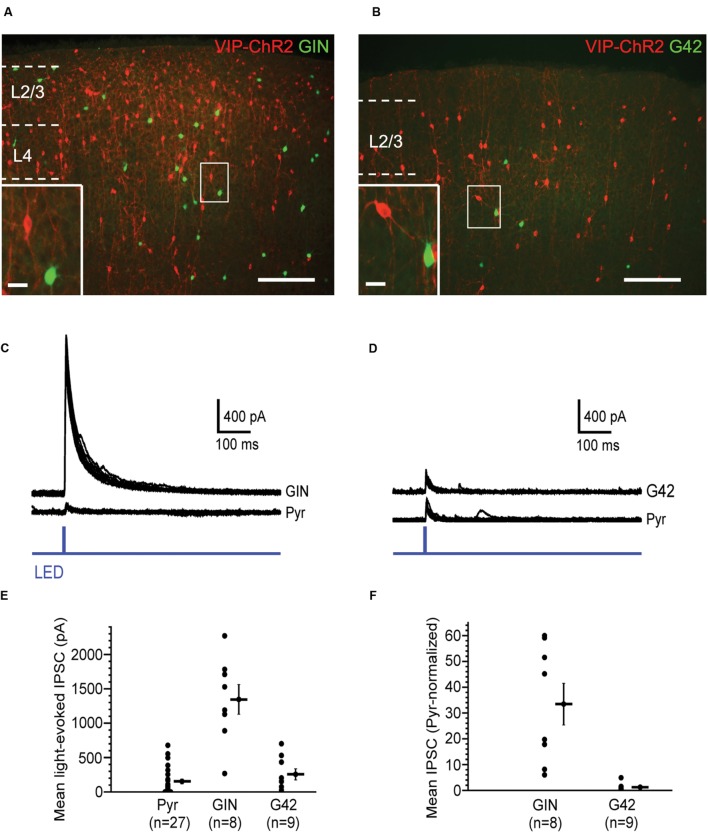
**Vasoactive intestinal peptide (VIP) cells strongly inhibit SOM cells.** **(A)** Fluorescent image of barrel cortex of GIN mouse (endogenous GFP in subset of SOM cells) crossed with VIP-Cre mouse, virally transfected with Cre-dependent, RFP-tagged ChR2; scale bar = 200 μm. Inset: expanded view of neighboring VIP cell (red) and GIN cell (green); scale bar = 20 μm. **(B)** Fluorescent image of barrel cortex of G42 mouse (endogenous GFP in subset of PV cells) crossed with VIP-Cre mouse, virally transfected with Cre-dependent, RFP-tagged ChR2; scale bar = 200 μm; dotted line denotes pia. Inset: expanded view of neighboring VIP cell (red) and G42 cell (green); scale bar = 20 μm. **(C)** Light-evoked IPSCs in a GIN cell and neighboring pyramidal cell in layer 2/3, in which VIP cells express ChR2. 10 traces overlaid for both GIN and pyramidal cell. **(D)** Light-evoked IPSCs in a G42 cell and neighboring pyramidal cell in layer 2/3, in which VIP cells express ChR2. 10 traces overlaid for both G42 and pyramidal cell. **(E)** Population data of mean light-evoked IPSCs in pyramidal, GIN, and G42 cells when VIP cells are optogenetically activated. IPSC_GIN_ > IPSC_Pyr_ (*P* = 1.1 × 10^-5^, Kruskal–Wallis test, Bonferroni correction), IPSC_GIN_ > IPSC_G42_ (*P* = 4.3 × 10^-4^, Kruskal–Wallis test, Bonferroni correction). **(F)** Population data of mean light-evoked IPSCs in GIN, and G42 cells, normalized to mean light-evoked IPSCs in pyramidal cells in same slice, when VIP cells are optogenetically activated, normIPSC_GIN_ > normIPSC_G42_ (*P* = 4.4 × 10^-4^, Mann–Whitney test).

Vasoactive intestinal peptide cells, another major NFS, PV-negative interneuron subtype, are particularly abundant in cortical layer 2/3 (Lee et al., 2010; Xu et al., 2010). Optogenetic activation of VIP cells evokes strong GABAergic responses in SOM cells, but much weaker responses in PV cells and pyramidal cells (Lee et al., 2013; Pfeffer et al., 2013; Pi et al., 2013). We confirmed that VIP cells in layer 2/3 preferentially target SOM cells, with much weaker targeting of PV cells and pyramidal cells (**Figure 3**). We crossed transgenic GIN mice, which express GFP in a subpopulation of SOM cells in layer 2/3, and transgenic G42 mice, which express GFP in a subpopulation of PV cells in layer 2/3, with VIP-Cre mice and injected barrel cortex of the progeny of these crosses with virus carrying a Cre-dependent, RFP-tagged ChR2 construct. We used the fluorescence of GIN and G42 cells to target them for recording. Using whole-cell micropipettes with a cesium-based internal solution, we voltage-clamped these cells at 0 mV to isolate IPSCs. In the presence of blockers of fast glutamatergic transmission, the ChR2-expressing VIP cell population was photostimulated with 5-ms-long single light pulses and the average peak (of 10 stimuli) of the light-evoked IPSC (relative to baseline) in the recorded GIN or G42 was calculated. In all slices used, we also recorded from pyramidal cells (2–4 in a given slice) within ∼100 μm of the recorded GFP-positive interneurons. The mean peak of the VIP-cell-evoked IPSC in GIN cells was 1346 pA, while the values in G42 cells and pyramidal cells were much lower: 256 and 154 pA, respectively (**Figure 3E**). We normalized the mean IPSC in each interneuron to the mean IPSC of the neighboring pyramidal cells in the same slices. VIP-cell-evoked IPSCs in GIN (SOM) cells were about 33 times greater than IPSCs of neighboring pyramidal cells, while IPSCs in G42 (PV) cells were similar in size to those of pyramidal cells (**Figure 3F**).

Thus, consistent with previous studies (Lee et al., 2013; Pfeffer et al., 2013; Pi et al., 2013), we conclude that GABAergic synapses from VIP cells are highly selective for SOM cells, and much less selective for PV and pyramidal cells.

### VIP Cells Do Not Control the Spiking Output of Pyramidal Cells during Up States

Recent studies in awake animals suggest that activation of VIP cells, either directly by optogenetic stimulation or indirectly via afferent pathways that strongly recruit them, leads to disinhibition in layer 2/3 pyramidal cells, enhancing their spiking output (Pi et al., 2013; Fu et al., 2014; Reimer et al., 2014; Zhang et al., 2014; Karnani et al., 2016a,b). We wondered whether the spontaneous spiking activity of VIP cells during Up states (Neske et al., 2015) might also serve to disinhibit layer 2/3 pyramidal cells. To test this, we expressed Arch in VIP cells and optically silenced them during Up states.

**FIGURE 4:**
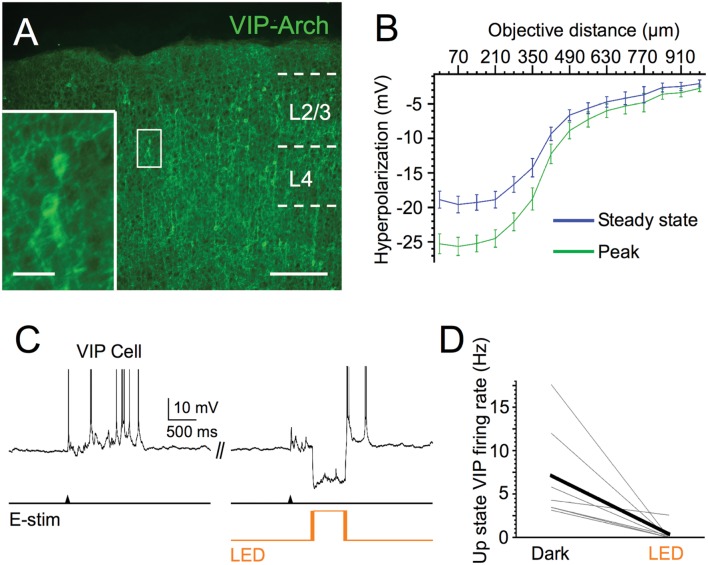
**Efficacy of Arch expression in VIP cells.** **(A)** Fluorescent image of Arch-EYFP-expressing VIP cells in superficial layers of barrel cortex; scale bar = 200 μm. Inset: expanded view of 2 Arch-expressing VIP cells, scale bar = 20 μm. **(B)** Light-evoked hyperpolarization (500 ms pulse duration) of Arch-expressing VIP cells in layer 2/3 as 40x immersion objective is moved away from the recorded cell in 70-μm increments (*n* = 6 cells). Peak and steady-state values are measured as in **Figures 1B,C**. **(C)** Voltage trace of example VIP cell during photostimulation. **(D)** Light stimuli significantly decrease spiking output of layer 2/3 Arch-expressing VIP cells during evoked Up states (*n* = 7 cells; Dark: 7.1 ± 2.1 Hz, LED: 0.4 ± 0.4 Hz, *P* = 0.022, paired *t*-test).

We first verified the efficacy of Arch in VIP cells. Light pulses centered on Arch-expressing VIP cells induced mean hyperpolarizations of -25.3 mV at peak and -18.9 mV at steady state (**Figure 4**). We also estimated the spatial extent of the light-evoked hyperpolarization by moving the objective away from the recorded cells in 70 μm increments. Light-evoked responses fell off rapidly after ∼270 μm, the radius of the light spot, yet hyperpolarizations remained detectable up to ∼1 mm from the cells (**Figure 4B**). Photostimuli completely silenced the Up state spiking of the majority (*n* = 5 of 7) of Arch-expressing VIP cells, decreasing the mean rate from 7.1 to 0.4 Hz (**Figure 4D**). Thus, photostimuli were effective in silencing VIP cells during Up states.

**FIGURE 5:**
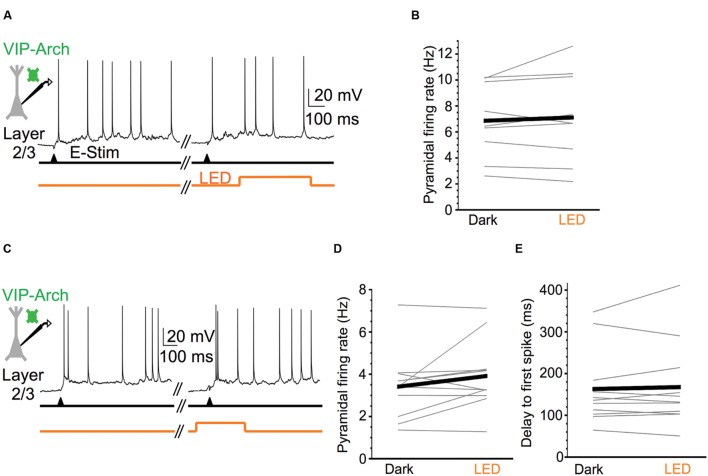
**Silencing VIP cells during Up states does not affect pyramidal cell excitability.** **(A)** Representative Up state membrane potential trace of a layer 2/3 pyramidal cell with and without optogenetic stimulation of Arch-expressing VIP cells in layer 2/3. **(B)** Population data demonstrating layer 2/3 pyramidal cell Up state firing rates do not significantly change when VIP cells are silenced (*n* = 9 cells; Dark: 6.9 ± 0.9 Hz, LED: 7.1 ± 1.2, *P* = 0.47, paired *t*-test). **(C)** As in **(A)**, but for a layer 2/3 pyramidal cell in which light stimuli were turned on before Up state onset and remained on for the first 250 ms of the Up state. **(D)** Population data demonstrating layer 2/3 pyramidal cell Up state firing rates do not significantly change when VIP cells are silenced during Up state onset (*n* = 10 cells, different set of neurons than in **B**; Dark: 3.4 ± 0.5 Hz, LED: 3.9 ± 0.5 Hz, *P* = 0.14, paired *t*-test). **(E)** As in **(D)**, but population data are delays from electrical stimulation to firing of the first action potential during an Up state (*n* = 10 cells, same set of neurons as in **D**; Dark: 163.0 ± 27.2 ms, LED: 167.6 ± 40.0 ms, *P* = 0.56, paired *t*-test).

We next tested the hypothesis that silencing VIP cells would decrease the firing rate of pyramidal cells during Up states, since such a manipulation would release SOM cells from the strong inhibition provided by VIP cells, leading to a net increase in the inhibition of pyramidal cells. Surprisingly, silencing VIP cells during Up states did not significantly affect the firing rates of layer 2/3 pyramidal cells (mean firing rates in control vs. light stimuli: 6.9 vs. 7.1 Hz; **Figures 5A,B**).

Since VIP cells are more active earlier in Up states (firing rate > 15 Hz) than later (∼5 Hz; Neske et al., 2015), we wondered whether VIP cells might only affect pyramidal cells during the beginning of Up states. To test this, we changed the stimulation protocol such that the LED turned on 100 ms before the electrical stimuli that evoked Up states (**Figure 5C**). The LED remained on for 250 ms after the electrical stimulus, and we analyzed pyramidal cell firing rates in this temporal window. This VIP cell silencing protocol did not significantly affect pyramidal cell firing rates (control vs. light: 3.4 vs. 3.9 Hz; **Figure 5D**), or the delay between the electrical stimulus and the first spike fired by pyramidal cells (control vs. light: 163.0 vs. 167.6 ms; **Figure 5E**; note that for two pyramidal cells the delay to the first spike fell outside the 250 ms analysis window. We nevertheless included them in the data set).

We conclude that the spontaneous firing of VIP cells during Up states does not significantly contribute to the control of spiking output in layer 2/3 pyramidal cells.

### Strong Activation of VIP Cells Does Not Affect the Spiking Output of Pyramidal Cells during Up States

**FIGURE 6:**
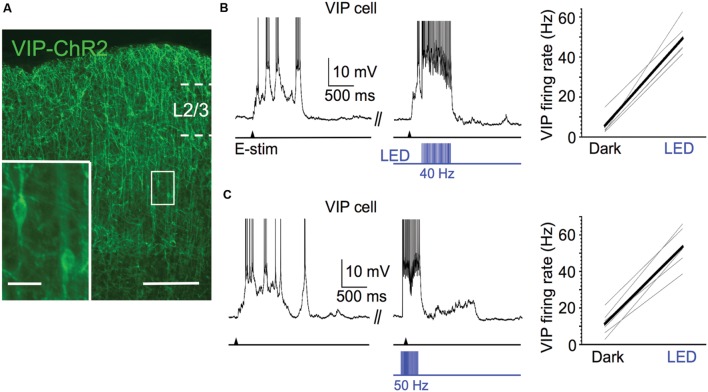
**Efficacy of ChR2 expression in VIP cells.** **(A)** Fluorescent image of ChR2-EYFP-expressing VIP cells in superficial layers of barrel cortex; scale bar = 200 μm. Inset: expanded view of 2 ChR2-expressing VIP cells, scale bar = 20 μm. **(B)** 40 Hz light pulse trains (pulse width = 5ms) effectively entrain spiking of ChR2-expressing VIP cells. Voltage trace of example VIP cell is shown to left (action potentials truncated). Shown to right are population data (*n* = 6 cells; Dark: 5.7 ± 1.9 Hz, LED: 49.3 ± 3.1 Hz, *P* = 4.4 × 10^-6^, paired *t*-test). **(C)** As in **(A)**, but light pulse trains are 50 Hz and given 100 ms before electrical stimulation and left on for 250 ms after electrical stimulation. Voltage trace of example VIP cell is shown to left (action potentials truncated). Shown to right are population data (*n* = 6 cells; Dark: 11.4 ± 2.7 Hz, LED: 53.6 ± 4.1 Hz, *P* = 4.3 × 10^-6^, paired *t*-test).

Several studies in awake mice have shown that exogenous excitatory drive to VIP cells disinhibits pyramidal cells, enhancing their firing rate (Pi et al., 2013; Fu et al., 2014; Reimer et al., 2014; Zhang et al., 2014; Karnani et al., 2016a,b). While the results in the previous section suggest that the spontaneous firing of VIP cells during Up states does not affect the excitability of pyramidal cells, we wondered whether strong activation of VIP cells during Up states might enhance the excitability of pyramidal cells through disinhibition. To test this, we expressed ChR2 in VIP cells (**Figure 6A**) and photostimulated them during Up states with 40 Hz light pulse trains (pulse width of 5 ms). These stimuli were effective in entraining the spiking of VIP cells during Up states near or above 40 Hz (**Figure 6B**; firing frequencies above 40 Hz were often due to burst-spiking during the 5 ms light pulse). During Up states, optical stimulation of ChR2-expressing VIP cells increased their mean firing rates from 5.7 to 49.3 Hz.

**FIGURE 7:**
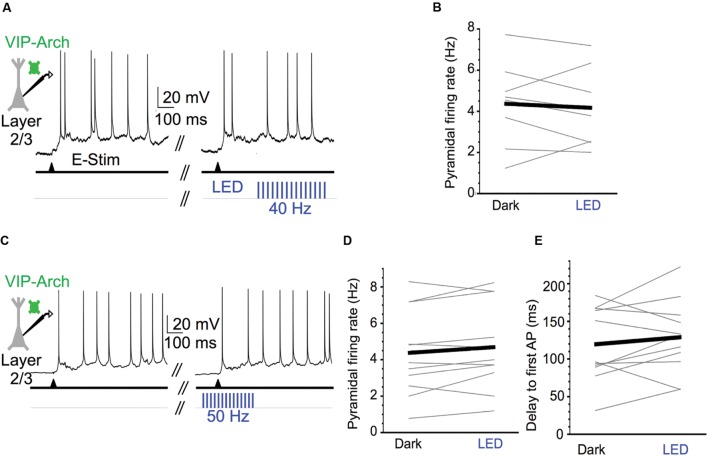
**Activating VIP cells during Up states does not affect pyramidal cell excitability.** **(A)** Representative Up state membrane potential trace of a layer 2/3 pyramidal cell with and without optogenetic stimulation of Arch-expressing VIP cells in layer 2/3. **(B)** Population data demonstrating layer 2/3 pyramidal cell Up state firing rates do not significantly change when VIP cells are activated at 40 Hz (*n* = 8 cells; Dark: 4.4 ± 0.7 Hz, LED: 4.2 ± 0.7 Hz, *P* = 0.12, paired *t*-test). **(C)** As in **(A)**, but for a layer 2/3 pyramidal cell in which 50 Hz light stimuli were turned on before Up state onset and remained on for the first 250 ms of the Up state. **(D)** Population data demonstrating layer 2/3 pyramidal cell Up state firing rates do not significantly change when VIP cells are activated during Up state onset (*n* = 11 cells, different set of neurons than in **B**; Dark: 4.4 ± 0.7 Hz, LED: 4.7 ± 0.7 Hz, *P* = 0.60, paired *t*-test). **(E)** As in **(D)**, but population data are delays from electrical stimulation to firing of the first action potential during an Up state (*n* = 11 cells, same set of neurons as in **D**; Dark: 119.5 ± 14.8 ms, LED: 128.9 ± 14.8 ms, *P* = 0.34, paired *t*-test).

We next tested the effect of strong VIP cell activation on layer 2/3 pyramidal cell spiking during Up states. We predicted that activation of VIP cells would enhance the spiking output of pyramidal cells. The light pulse frequency was increased from 40 to 50 Hz in this case since, primarily due to burst-firing, some VIP cells can exhibit firing rates >30 Hz during the initial portion of the Up state (Neske et al., 2015). The 50 Hz stimuli effectively entrained VIP cells during the initial portion of the Up state near or above 50 Hz (**Figure 6C**). Unexpectedly, however, we found that photostimulating the VIP cells had no effect on the mean Up state firing rates of pyramidal cells (control vs. light: 4.4 vs. 4.2 Hz; **Figures 7A,B**). Light pulses that began 100 ms before evoking an Up state and continued for its first 250 ms also had no effect on pyramidal cell firing (control vs. light: 4.4 vs. 4.7 Hz; **Figures 7C,D**) and did not significantly change the delay between electrical stimulation and the first spike during an Up state (control vs. light: 119.5 vs. 128.9 ms; **Figure 7E**).

We conclude that the firing of VIP cells, whether it occurs spontaneously or is strongly driven by photostimulation, does not control the spiking of layer 2/3 pyramidal cells during Up states. This is remarkable given previous results in awake animals.

## Discussion

We studied the contributions of SOM and VIP cell activity to the excitability of pyramidal cells during Up states in mouse barrel cortex. Optogenetic inactivation of the SOM cell population significantly enhanced the spike output of pyramidal cells. This suggests that the natural spiking of SOM cells during Up states provides significant inhibitory modulation of pyramidal cells. Optogenetic inactivation of VIP cells, however, did not significantly change spiking in layer 2/3 pyramidal cells. Furthermore, despite demonstrably powerful innervation of SOM cells by VIP cells, strong optogenetic activation of VIP cells also did not significantly alter the spiking of layer 2/3 pyramidal cells. Thus, during Up states, VIP cells minimally contribute to the excitability of pyramidal cells both in baseline conditions and when VIP cells are strongly recruited.

Diverse GABAergic interneurons provides inhibitory balance to recurrent excitation during cortical network activity. FS-PV cells undoubtedly provide the preponderance of inhibition in active cortical networks, given their abundance among GABAergic cells (Lee et al., 2010; Xu et al., 2010; Rudy et al., 2011), high probability of connecting with local excitatory cells (Beierlein et al., 2003; Holmgren et al., 2003; Avermann et al., 2012), and high spontaneous firing rate (Hasenstaub et al., 2005; Fanselow and Connors, 2010; Gentet et al., 2010, 2012; Neske et al., 2015). Furthermore, in certain cortical areas, such as entorhinal cortex (Tahvildari et al., 2012; Neske et al., 2015), FS-PV cells are virtually the only active GABAergic cell type during Up states. However, in the barrel cortex and perhaps other sensory cortices, all major neurochemically defined interneuron subtypes exhibit spontaneous firing rates comparable to (in layer 5) or higher than (in layer 2/3) neighboring pyramidal cells (Gentet et al., 2010, 2012; Neske et al., 2015). Here, we showed that silencing the SOM cell population during Up states significantly enhanced the excitability of neighboring pyramidal cells, suggesting SOM cells play an important role in the regulation of Up states. This is consistent with previous work demonstrating that optogenetic silencing of SOM cells enhances excitability of pyramidal cells (Gentet et al., 2012; Xu et al., 2013). A recent study suggested that the activity of SOM cells decreases release probability in glutamatergic pyramidal-to-pyramidal synapses via activation of presynaptic GABA_B_ receptors (Urban-Ciecko et al., 2015). Thus, SOM cell activity may affect pyramidal cell excitability by both post- and pre-synaptic mechanisms.

The inability of VIP cells to influence the Up state spiking of layer 2/3 pyramidal cells was unexpected. Several recent studies demonstrated the disinhibitory effects of VIP cells in awake animals in multiple sensory cortices (Pi et al., 2013; Fu et al., 2014; Reimer et al., 2014; Zhang et al., 2014; Karnani et al., 2016a). While awake animals and the active cortical slice are obviously quite different experimental preparations, it is important to consider the cellular and network properties that allow activation of VIP cells to disinhibit pyramidal cells in certain network states, but not others.

One factor that could determine whether VIP cell activation is disinhibitory is the relative firing rates of FS-PV cells vs. NFS-PV-negative cells, particularly SOM cells. In the anesthetized animal, and likely also during quiescent sleep, the Up state firing rates of fast-spiking PV cells are usually twice that of regular-spiking, putative pyramidal cells (Hasenstaub et al., 2005; Haider et al., 2006; Massi et al., 2012). In L2/3 of mouse barrel cortex during quiet wakefulness, this dichotomy of cell-type-specific firing rates is even more pronounced due to the low firing rates of pyramidal cells (Gentet et al., 2010, 2012). The much higher firing rates of FS-PV cells relative to pyramidal cells and NFS-PV-negative cells is reproduced during the slow oscillation of barrel cortex *in vitro* (Neske et al., 2015).

During such periods, in which FS-PV cells undoubtedly provide the majority of synaptic inhibition to the cortical network, activation of VIP cells, and subsequent inhibition of SOM cells, might have a negligible effect on the excitability of pyramidal cells since the share of inhibition onto pyramidal cells contributed by SOM cells is probably low. In effect, during a PV cell-dominated state, such as the Up states of quiescent sleep and quiet wakefulness, the spike-generating mechanism of pyramidal cells might not be able to detect decreases in the spontaneous firing rates of SOM cells. While complete or nearly complete silencing of the SOM cell population indeed does cause significant changes in the spiking output of pyramidal cells during Up states (see Results and Gentet et al., 2012; Xu et al., 2013), GABAergic inputs from VIP cells onto SOM cells, while strong, are unlikely to be as powerful as the direct, light-evoked activation of hyperpolarizing opsins. We did not, however, record from SOM cells during activation of VIP cells during Up states. Thus, we suggest that in a network state in which the share of synaptic inhibition provided by PV cells is very high, VIP cell activation is unlikely to elicit disinhibition in pyramidal cells since the share of inhibition provided by SOM cells is relatively low. This prediction could be tested with “two-color” optogenetic experiments, in which hyperpolarizing opsins could be expressed in PV cells, while depolarizing opsins could be expressed in VIP cells directly or in the “feedback” axons that preferentially target VIP cells. The disinhibitory effect of VIP cells could then be compared between control conditions and conditions in which PV cell activity is optogenetically diminished.

During cortical state changes from quiet wakefulness to active behavior, the relative firing rates of FS-PV cells and NFS-PV-negative cells change dramatically. While the firing rate of PV cells decreases by approximately 50% from quiet wakefulness to active behavior (from ∼11 to ∼4 Hz), the firing rate of NFS interneurons increases by approximately 50% (from ∼2 to ∼5 Hz; Gentet et al., 2010), resulting in an effective equalization of firing rates between FS and NFS interneurons during active behavior. These divergent, cell-type-specific changes in excitability from quiet wakefulness to active behavior might be due to the different effects of neuromodulators: potentiating for NFS-PV-negative cells (Beierlein et al., 2000; Férézou et al., 2002; Fanselow et al., 2008; Lee et al., 2010; Arroyo et al., 2012; Chen et al., 2015) and suppressing for FS-PV cells (Kruglikov and Rudy, 2008; Puig et al., 2010; Yamamoto et al., 2010; Lladó-Pelfort et al., 2012). While SOM cells, unlike other NFS interneurons, also decrease their firing rates from quiet wakefulness to active behavior (from ∼6 to ∼2 Hz; Gentet et al., 2012), the difference in firing rate between SOM cells and PV cells during active behavior (∼2 Hz) is still smaller than the difference in firing rate between these cells during quiet wakefulness (∼5 Hz). Thus, the relative contributions of inhibition onto pyramidal cells provided by PV and SOM cells may be more equalized during active behavior compared to quiescent behavioral states. It follows that since SOM cells contribute a greater share of the inhibitory conductance in pyramidal cells during active behavior, changes in SOM cell firing rates might be more detectable by pyramidal cells in this cortical state.

Another consideration when determining the disinhibitory function of VIP cells is the synaptic interaction between SOM cells and PV cells. In multiple cortical areas, it has been established that SOM cells innervate PV cells (Gibson et al., 1999; Beierlein et al., 2003; Hu et al., 2011; Cottam et al., 2013; Pfeffer et al., 2013; Xu et al., 2013). If the primary post-synaptic target of VIP cells is SOM cells, one might expect that VIP cell activation would enhance the excitability of PV cells due to their release from SOM-cell-dependent inhibition. If this is the case, then the effect of VIP cell activation on pyramidal cell excitability would depend upon the net effect of increased PV cell activity and decreased SOM cell activity. It is possible that a decrease in SOM cell activity will lead to an increase in PV cell activity. During activation of VIP cells, even if the average increase in PV cell activity is smaller than the average decrease in SOM cell activity, the higher abundance, perisomatic synaptic targeting, and stronger unitary synaptic properties of PV cells might allow these cells to compensate for the decreased inhibition from SOM cells. Since we did not record from PV cells during optogenetic activation of VIP cells during Up states, we cannot rule out this scenario as an explanation for why we did not observe changes in pyramidal cell excitability. VIP-cell-based disinhibition of PV cells might depend on neuromodulatory tone. In particular, if the SOM cell-to-PV cell inhibitory synapse is suppressed while the SOM-to-pyramidal cell inhibitory synapse is unchanged or potentiated, a disinhibitory action of VIP cell activation would be more likely.

The contributions of distinct inhibitory interneuron subtypes during recurrent cortical activation likely also depend on the short-term dynamics of the synapses by which these subtypes are integrated into the cortical network. The dynamics of excitatory and inhibitory cortical synapses substantially vary depending on the identity of the post- and pre-synaptic cell. Excitatory and inhibitory synapses involving pyramidal and PV cells are depressing, excitatory synaptic inputs onto SOM cells are facilitating, and the inhibitory synaptic inputs from SOM cells onto pyramidal and PV cells vary from relatively flat dynamics to weakly facilitating (Markram et al., 1998; Reyes et al., 1998; Gibson et al., 1999; Beierlein et al., 2003; Ma et al., 2012). A recent study elucidated the dynamics of the synapses associated with the pyramidal-SOM-VIP cell circuit: excitatory synapses from pyramidal cells to VIP cells are strongly depressing, but inhibitory synapses from VIP to SOM cells and SOM cells to VIP cells are strongly facilitating (Karnani et al., 2016b). The functional interplay among the diverse cell-type-specific synaptic dynamics in the cortical circuit are difficult to predict and will undoubtedly be challenging to disentangle experimentally. Furthermore, in addition to the modulation of intrinsic excitability, the dynamic neuromodulatory tone associated with cortical state fluctuations also modulates excitatory and inhibitory synaptic dynamics. It will be important for future studies to clarify, in particular, the possible state-dependent modulation of the synaptic dynamics in the pyramidal-SOM-VIP circuit, since this circuit appears to play a particularly important role during waking states.

## Author Contributions

GN and BC designed research. GN conducted experiments and analyzed data. GN and BC wrote the manuscript.

## Conflict of Interest Statement

The authors declare that the research was conducted in the absence of any commercial or financial relationships that could be construed as a potential conflict of interest.
